# Supplementary material for: Resolving the full spectrum of human genome variation using Linked-Reads
Source: Genome Res. 2019 Apr;29(4):635–45. doi: 10.1101/gr.234443.118 (PMC6442396; doi:10.1101/gr.234443.118)
Supplement: Supplemental Material [file supp_gr.234443.118_Supplemental_longranger-2.2.zip › longranger-2.2.tar.bz2!/lr2.2-public/lib/stan/cmdstan/stan/doc/api/html/functions_vars_a.html]

Stan: Class Members - Variables


|  |  |
| --- | --- |
|  | Stan  2.5.0  probability, sampling & optimization |


- Main Page
- Related Pages
- Namespaces
- Classes
- Files

- Class List
- Class Index
- Class Hierarchy
- Class Members

- All
- Functions
- Variables
- Typedefs
- Enumerator
- Related Functions

- \_
- a
- b
- c
- d
- e
- f
- g
- h
- i
- k
- l
- m
- n
- o
- p
- q
- r
- s
- t
- u
- v
- w
- x
- y
- z


All Classes Namespaces Files Functions Variables Typedefs Enumerations Enumerator Friends Macros Pages

### - a -

- ad\_
  : stan::agrad::op\_ddv\_vari
  , stan::agrad::op\_dv\_vari
  , stan::agrad::op\_dvd\_vari
  , stan::agrad::op\_dvv\_vari
- adapt\_base\_window\_
  : stan::mcmc::windowed\_adaptation
- adapt\_flag\_
  : stan::mcmc::base\_adapter
- adapt\_init\_buffer\_
  : stan::mcmc::windowed\_adaptation
- adapt\_next\_window\_
  : stan::mcmc::windowed\_adaptation
- adapt\_term\_buffer\_
  : stan::mcmc::windowed\_adaptation
- adapt\_window\_counter\_
  : stan::mcmc::windowed\_adaptation
- adapt\_window\_size\_
  : stan::mcmc::windowed\_adaptation
- adaptation
  : stan::io::stan\_csv
- adj\_
  : stan::agrad::vari
- algorithm
  : stan::io::stan\_csv\_metadata
- all\_constant
  : stan::agrad::OperandsAndPartials< T1, T2, T3, T4, T5, T6, T\_return\_type >
- all\_partials
  : stan::agrad::OperandsAndPartials< T1, T2, T3, T4, T5, T6, T\_return\_type >
- all\_varis
  : stan::agrad::OperandsAndPartials< T1, T2, T3, T4, T5, T6, T\_return\_type >
- alpha0
  : stan::optimization::LSOptions< Scalar >
- append\_samples
  : stan::io::stan\_csv\_metadata
- arg\_decl\_r
  : stan::gm::functions\_grammar< Iterator >
- arg\_decls\_
  : stan::gm::function\_decl\_def
- arg\_decls\_r
  : stan::gm::functions\_grammar< Iterator >
- arg\_type\_
  : stan::gm::arg\_decl
- args\_
  : stan::gm::array\_literal
  , stan::gm::distribution
  , stan::gm::fun
- args\_r
  : stan::gm::term\_grammar< Iterator >
- array\_dims\_r
  : stan::gm::bare\_type\_grammar< Iterator >
- assignment\_r
  : stan::gm::statement\_grammar< Iterator >
- avi\_
  : stan::agrad::op\_v\_vari
  , stan::agrad::op\_vd\_vari
  , stan::agrad::op\_vdd\_vari
  , stan::agrad::op\_vdv\_vari
  , stan::agrad::op\_vv\_vari
  , stan::agrad::op\_vvd\_vari
  , stan::agrad::op\_vvv\_vari


---

[ Stan Home Page ]

*© 2011–2014,
Stan Development Team.*
